# Roles of HLA-G in the Maternal-Fetal Immune Microenvironment

**DOI:** 10.3389/fimmu.2020.592010

**Published:** 2020-10-22

**Authors:** Xiuxiu Xu, Yonggang Zhou, Haiming Wei

**Affiliations:** ^1^Hefei National Laboratory for Physical Sciences at Microscale, Division of Molecular Medicine, The CAS Key Laboratory of Innate Immunity and Chronic Disease, School of Life Sciences, University of Science and Technology of China, Hefei, China; ^2^Institute of Immunology, University of Science and Technology of China, Hefei, China; ^3^The First Affiliated Hospital of USTC, Division of Life Sciences and Medicine, University of Science and Technology of China, Hefei, China

**Keywords:** human leukocyte antigen G, pregnancy, extravillous trophoblasts, immunology, natural killer cells, spiral artery remodeling, fetal development

## Abstract

During pregnancy, the maternal uterus and fetus form a special microenvironment at the maternal-fetal interface to support fetal development. Extravillous trophoblasts (EVTs), differentiated from the fetus, invade into the decidua and interact with maternal cells. Human leukocyte antigen (HLA)-G is a non-classical MHC-I molecule that is expressed abundantly and specifically on EVTs in physiological conditions. Soluble HLA-G (sHLA-G) is also found in maternal blood, amniotic fluid, and cord blood. The abnormal expression and polymorphisms of HLA-G are related to adverse pregnancy outcomes such as preeclampsia (PE) and recurrent spontaneous abortion (RSA). Here we summarize current findings about three main roles of HLA-G during pregnancy, namely its promotion of spiral artery remodeling, immune tolerance, and fetal growth, all resulting from its interaction with immune cells. These findings are not only of great significance for the treatment of pregnancy-related diseases but also provide clues to tumor immunology research since HLA-G functions as a checkpoint in tumors.

## Introduction

The HLA-G gene was first discovered in 1982 (1) and was denominated HLA-G in 1990 (2). HLA-G is a class I histocompatibility antigen, but unlike the classical class I major histocompatibility complex (MHC-I) HLA-A, HLA-B, and HLA-C genes, HLA-G displays limited polymorphism. Seven different HLA-G mRNA transcripts have been identified, with this variety attributed to alternative splicing of its seven exons. HLA-G2, G3, and G4 transcripts are translated into membrane-binding isoforms; HLA-G5, G6, G7 into soluble HLA-G (sHLA-G) isoforms; and HLA-G1 into both types of isoforms (3–6).

HLA-G appears to be especially relevant to pregnancy. After ovulation, the uterine stromal fibroblasts of the endometrium differentiate into decidual cells. In addition, uterine spiral arteries are formed. In early pregnancy, steroid hormones, progesterone, and β-estradiol act on maternal vascular endothelial cells and increase vascular permeability, promoting angiogenesis (7–9). Immune cells, especially natural killer (NK) cells, are recruited though maternal vessels. Extravillous trophoblasts (EVTs) of the embryo invade into the decidua and replace the endothelial cells (10). Together, these processes remodel spiral arteries and form the maternal-fetal interface to support the provision of oxygen and nutrients for fetal development.

HLA-G has been reported to be expressed on the surfaces of preimplantation embryos (11–13) and EVTs (14–16), while sHLA-G has been detected in culture medium of *in vitro*-fertilized (IVF) embryos (17, 18), maternal blood (19–21), amniotic fluid (22, 23), and cord blood (24, 25). HLA-G plays critical roles in the remodeling of spiral arteries, fetal development, and immune tolerance (10).

Considering these findings involving HLA-G, it is not surprising to also find relationships between HLA-G and complications associated with pregnancy. Specifically, HLA-G gene polymorphisms and decreased levels of sHLA-G have been found to be related to embryo implantation failure (18, 26–30), recurrent spontaneous abortion (31–36), placental abruption (37) and pre-eclampsia (38–48). For example, a multicenter study showed that the presence of soluble HLA-G in the culture medium of the embryo is significantly associated with increased pregnancy rates after assisted reproduction technique (ART) (26). Furthermore, Nowak et al. observed that soluble HLA-G level in the serum of patients is correlated with pregnancy outcome after ART. In addition, they revealed significantly association of G-C-ins (-725G>C SNP in the promoter region of HLA-G) haplotypes with infertility (27). HLA-G plasma level in women with placental abruption was significantly decreased (37). It is therefore critical to achieve a comprehensive understanding of the roles of HLA-G in pregnancy in general, and in maternal-fetal interactions specifically. Herein, we review three such major identified roles.

## HLA-G Promotes the Remodeling of Spiral Artery

Fetal development in the uterus requires nutrients and oxygen provided by the maternal blood. Spiral artery remodeling is essential for accelerating and stabilizing placental blood flow during pregnancy. This remodeling begins with decidua-associated remodeling and involves the initial swelling and disorganization of the vascular smooth muscle of spiral artery. Next the vascular endothelium becomes liquefied and the elastic membrane disintegrates. These changes are mainly induced by angiogenic growth factors, which have been found to be produced by decidual NK cells and macrophages (49–53).

NK cells constitute less than 20% of human peripheral lymphocytes, but account for about 70% of lymphocytes in the first-trimester decidua (54), and are hence the most abundant lymphocytes in the early maternal-fetal interface. NK cells recognize MHC-I proteins on target cells by expressing receptors such as killer cell immunoglobulin-like receptors (KIRs). And then NK cells decide whether to kill target cells upon signals from inhibitory or activating receptors.

KIR2DL4 (also called CD158d), a member of the KIR family, has a structure, localization, and function that differs from that of other KIRs (55–57). KIR2DL4 is expressed by NK cells and activates the production of IFN-γ but does not promote cytotoxicity of resting peripheral NK cells (58, 59). One variant of KIR2DL4, 9A is unstable on the surface because of its truncated cytoplasmic tail (60). In addition, KIR2DL4 has been reported to be mainly localized in intracellular endosomes containing Rab5 (61) and this localization requires the Ig domain of KIR2DL4. The localization and function in endosomes is specific to KIR2DL4. Because the expression of other members of the KIR family on the cell surface is very high.

The ligand of KIR2DL4 is HLA-G. Rajagopalan et al. (57) reported that soluble KIR2DL4, a fusion protein composed of the extracellular region of KIR2DL4 and the IgG Fc region, binds to LCL 721.221 cells that express HLA-G but not HLA-Cw3 or HLA-B7. Furthermore, soluble HLA-G or HLA-G on the surface of target cells was observed to be endocytosed into vesicles of NK cells after interacting with KIR2DL4. Soluble HLA-G has been shown to activate the secretion of proangiogenic/proinflammatory cytokines and chemokines (i.e., IL-6, IL-1β, IL-8, IL-23, MIP-1-α, and MIP-3-α) by NK cells. In addition, the secretion of IL-8 was shown to require the cytoplasmic tail of KIR2DL4 (61). Rajagopalan et al. (62) later described the molecular mechanism involved in this process. In NK cells, KIR2DL4 was found to interact with DNA damage signaling kinase DNA-PKcs and trigger phosphorylation of Akt at position Ser^473^ following stimulation with soluble HLA-G. Phosphorylated Akt activates the NF-κB pathway and hence results in the production of proinflammatory and proangiogenic cytokines (62). In addition, Rajagopalan et al. (63) identified a mutation of the TRAF6-binding motif in the KIR2DL4 cytoplasmic tail that caused decreased IL-8 secretion in transfected 293T cells. A co-immunoprecipitation assay using NK cells demonstrated an association between TRAF6 and KIR2DL4. And the TRAF6 binding site was shown to be required for the NF-κB signaling pathway. Specifically, KIR2DL4 recruits TRAF6, which in turn phosphorylates TAK1 at position T187. TAK1 participates in the production of IL-6, IFN-γ, CXCL1, and P2RX5 in NK cells (63).

**Figure 1:**
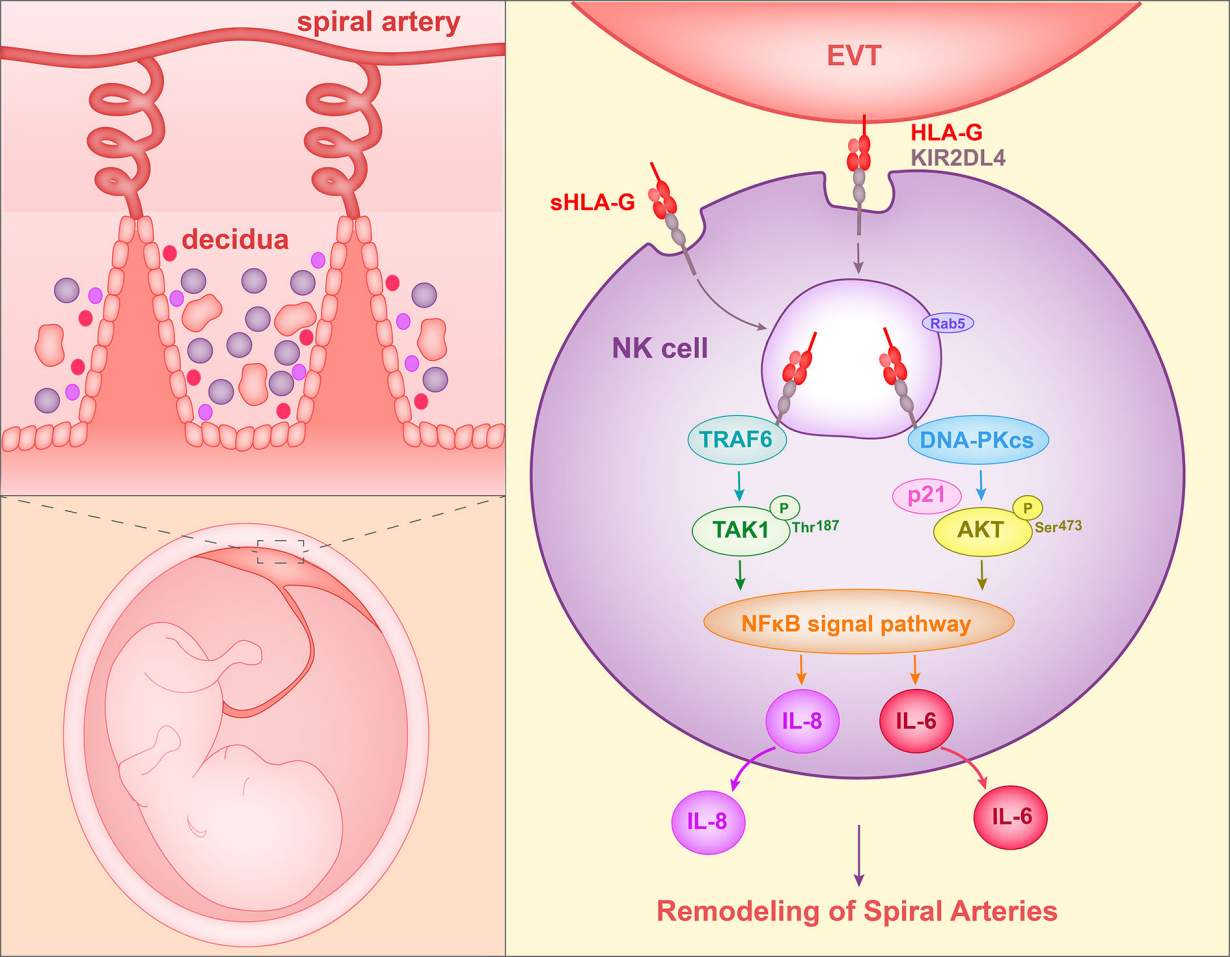
Proposed interaction of HLA-G with decidual NK cells to promote spiral artery remodeling. HLA-G on EVTs or sHLA-G secreted by EVTs binds to KIR2DL4 on NK cells, and HLA-G and KIR2DL4 are then endocytosed into Rab5^+^ endosomes of NK cells. The endocytosed KIR2DL4 binds to TRAF6, induces phosphorylation of TAK1 at Thr187 and activates the NF-κB pathway. In addition, KIR2DL4 interacts with DNA-PKcs, triggering phosphorylation of Akt at Ser473 and upregulating p21. Phosphorylated Akt then activates the NF-κB pathway and results in the expression of the senescence-associated secretory phenotype (SASP) and production of, for example, IL-6 and IL-8. The SASP promotes vascular permeability, angiogenesis and invasion of EVT.

When peripheral NK cells were stimulated with soluble HLA-G (sHLA-G) or KIR2DL4 agonist antibody, DNA-PKcs was activated and induced the expression of cyclin-dependent kinase inhibitor p21. At the same time, heterochromatin protein 1-γ (HP1-γ) was phosphorylated at position Ser-83. These events are related to cell senescence. NK cells activated by KIR2DL4 and sHLA-G acquired senescence features, in particular they became enlarged and showed increased β–galactosidase (SA-β-gal) activity. However, proliferation and apoptosis were not induced. Supernatants from peripheral NK cells stimulated with agonist antibody of KIR2DL4 could enhance HUVEC vascular permeability and tube formation. This observation is consistent with the stimulation by TNF-α and IL-1β (64). These data acquired by Long et al. showed that sHLA-G can stimulate the production of senescence-associated secretory phenotype (SASP) in NK cells by binding to KIR2DL4, and hence promote vascular permeability and angiogenesis (65). Thus, in this way, HLA-G can facilitate spiral artery remodeling (**Figure 1**).

KIR2DL4 mRNA has been detected in peripheral NK cells of every donor tested (66). Nonetheless, protein levels of KIR2DL4 were reported to be very low on the surface of resting peripheral NK cells and decidual NK cells (67). In addition, the investigations, which sHLA-G and KIR2DL4 are endocytosed into endosomes and induce secretion of proangiogenic cytokines that promote vascular permeability, were verified in peripheral NK cells. Peripheral NK cells are quite different from decidual NK cells, and hence whether this process and mechanism occur in decidual NK cells remains to be determined. Furthermore, Fu et al. (68) observed the expression of intracellular KIR2DL4 in first-trimester decidual NK cells. This observation is consistent with the findings that KIR2DL4 resides in endosomes of decidual NK cells.

HLA-G has been reported to be selectively expressed on the cell surface of extravillous trophoblasts (14, 15). In addition, Apps et al. (69) found a significant percentage of HLA-G expressed as a homodimer on the surfaces of trophoblast cells in first-trimester placenta. This HLA-G homodimer is disulphide-linked and β2m-associated (69, 70). LCL 721.221 cells that express homodimeric HLA-G could stimulate CD14^+^ decidual macrophages or decidual NK cells to secrete cytokines such as IL-6, IL-8, and TNF-α (53). This stimulation is activated when homodimeric HLA-G binds to immunoglobulin-like transcript 2 (ILT2, also called LILRB1) on macrophages or to KIR2DL4 on NK cells. Macrophages differentiated from peripheral monocytes could exhibit the phenotype of decidual macrophages when stimulated by soluble HLA-G5. These macrophages were observed to secrete more IL-6 and CXCL-1 and to induce trophoblast invasion (71). In addition, soluble HLA-G5 could also form a homodimer (69). Although, it remains to be determined whether sHLA-G in decidua and maternal serum exists as a homodimer or monomer.

sHLA-G induces the secretion of IL-8 in NK cells and homodimeric HLA-G on EVTs can stimulate decidual NK cells and macrophages to produce IL-8. Hanna et al. (72) found that decidual NK cells highly express IL-8, which can bind to CXCR1 and CXCR3. In addition, a transwell assay showed that IL-8 neutralizing antibody could reduce invasiveness of trophoblasts induced by decidual NK cells. Furthermore, neutralizing antibody to IL-8 could inhibit trophoblast invasion induced by decidual NK cells in Matrigel on nude mice. Therefore, IL-8 secreted by decidual NK cells was determined to participate in trophoblast invasiveness. In addition, a population of ILT2^hi^ pregnancy-trained decidual NK cells has been identified in repeated pregnancies. HLA-G-expressing LCL721.221 cells could enhance the secretion of more VEGFα in decidual NK cells of women with multigravid pregnancies. VEGFα secreted by decidual NK cells promotes vascularization (73).

In addition, HLA-G1^+^ APCs have been shown induce the differentiation of CD4^+^ peripheral T cells into suppressive CD4^+^ T cells (74). It has been reported that decidual Treg cells can increase trophoblast invasiveness. This effect is inhibited by anti-IL-10 neutralizing antibody and is increased by anti-TGF-β antibody (75).

The remodeling of spiral artery in women with pre-eclampsia is impaired (76). In patients with pre-eclampsia, the level of soluble HLA-G in serum was detected to be decreased whether in the first, second or third trimester of pregnancy (41, 50). In addition, HLA-G3 transcript was significantly reduced in the placenta of mild pre-eclampsia (42). Furthermore, the frequency of +14 bp/+14 bp genotype (14 bp insert in exon 8 of the HLA-G gene) in pre-eclampsia offspring was higher than in control offspring. In addition, fetal HLA-G*0106 in combination with maternal KIR2DL4*006 allele was reported to be associated with pre-eclampsia risk among multigravid pregnancies (51). Therefore, it will accelerate the treatment and prevention of preeclampsia to investigate the role of HLA-G in spiral artery remodeling.

## HLA-G Participates in the Formation of Maternal-Fetal Immune Tolerance

In the uterus, the fetus expresses paternal histocompatibility antigens, which are foreign antigens for the mother, yet the fetus is neither rejected nor attacked by the maternal immune system. This phenomenon is related to the specific immune tolerance microenvironment at the maternal-fetal interface. In the first trimester, immune cells account for up to 40% of the decidua. NK cells, macrophages, and T cells make up, respectively, about 70%, 15%–20%, and 5%–15% of decidual leukocytes (77). In addition, myeloid dendritic cells (DCs) have also been detected. Fetal-derived EVTs invade into the decidua and come into direct contact with maternal leukocytes. EVTs express classical MHC I HLA-C molecules and non-classical MHC I HLA-G and HLA-E molecules, and interact with leukocytes expressing receptors (such as KIR) to provide immune tolerance conditions (78).

NK cells are the most abundant leukocytes in the decidua during the first trimester. Expression of HLA-G has been suggested to protect NK-cell-sensitive cells from lysis by NK cell lines or decidual NK cells (79, 80). Moreover, NK cells in the maternal uterine blood were observed to not kill cytotrophoblast cells, whether the cytotrophoblast cells were isolated from the same mothers from whom the NK cells were derived or from other mothers. Cytotoxicity of NK cells was restored if an antibody that blocks both HLA-G and HLA-C was added into the co-culture medium. But an anti-HLA-C antibody did not reverse the protection against NK lysis. Therefore, HLA-G protected embryo-derived cytotrophoblast cells from being lysed by NK cells from the maternal uterine blood (81). However, cells overexpressing HLA-G have been reported to upregulate the surface expression of HLA-E, another nonclassical MHC molecule (82) since HLA-E can be loaded with HLA‐G‐derived peptide, the HLA-G leader sequence (83). Therefore, it is the HLA-E-NKG2A/CD94 interaction that inhibits cytotoxicity of NK cells to cell lines expressing HLA-G.

NK cells have been reported to express two HLA-G receptors: KIR2DL4 (57) and ILT2 (69). Co-culture of NK cell lines with HLA-G-expressing melanoma M8 cells restored the expression of KIR2DL4 or ILT2 on NK cells (84), and NK cells could use the trogocytosis to acquire HLA-G from tumor cells (85). Following co-culture with HLA-G1-expressing M8 cells or LCL 721.221 cells, IL-2-activated NK cells or IL-2-activated peripheral NK cells could acquire HLA-G1 on their cell surfaces and lose their cytotoxicity. Similarly, trogocytosis could transfer HLA-G to decidual NK cells from EVTs (86). While HLA-G mRNA was not detected, cell surface and intracellular HLA-G molecules were found in decidual NK cells after NK cells were co-cultured with EVTs. A transwell separation demonstrated NK cells obtaining HLA-G through direct cell contact. Therefore, decidual NK cells were shown to obtain HLA-G and then endocytose the HLA-G when in contact with EVTs. As mentioned above, HLA-G binds KIR2DL4 and is endocytosed by NK cells. Trogocytosis may play a role in NK cell tolerance.

Apps et al. (87) found that decidual NK cell degranulation was not affected when co-culturing with LCL 721.221 cells expressing HLA-G. The CD107a level in decidual NK cells was unchanged as a result of the stimulation of LCL 721.221 cells that express HLA-G. In addition, van der Meer et al. (88) reported that soluble HLA-G did not affect cytotoxicity of uterine mononuclear cells towards K562 cells and lytic activity of peripheral NK cells towards K562 cells was not affected by soluble HLA-G. However, soluble HLA-G could stimulate peripheral NK cells to produce interferon (IFN)-γ. Further, Poehlmann et al. (89) found that soluble HLA-G1 could inhibit cytotoxicity of term placentae NK cells towards K562 cells, NK cell sensitive cell lines, even when the NK cells were pre-stimulated with IL-2. And soluble HLA-G1 induced reduction of perforin in term placentae NK cells. Therefore, it is necessary to use a system of decidual NK cells and EVT to study the effects and underlying mechanism of HLA-G on decidual NK cells tolerance to EVTs. Du et al. (75) reported that lytic activity of decidual NK cells towards K562 cells was reduced when decidual NK cells were pre-cocultured with trophoblasts. Trophoblasts pre-incubated with Treg cells showed a greater downregulation effect, attributed to decidual CD4^+^CD25^+^ Treg cells upregulated HLA-G expression in the trophoblasts. Furthermore, a neutralizing antibody to HLA-G could rescue the cytotoxicity of decidual NK cells. Thus, Treg cells could promote the expression of HLA-G in trophoblasts and inhibit cytotoxicity of decidual NK cells, and hence facilitate the production of IL-4 and IL-10 by decidual NK cells. In addition, de Mendonça Vieira found that HLA-G expression on term placenta EVTs is higher than that on first trimester EVTs. Increased HLA-G could provide increased interaction with term pregnancy dNK cells through KIR2DL4 and ILT2. However, term pregnancy dNK cells showed increased degranulation capacity in response to PMA/ionomycin and K562 cells (90). Therefore, HLA-G may have different effects on dNK cells in different stages of pregnancy.

In first-trimester decidua, 10%–15% of leukocytes are T cells. This proportion has been shown to rise up to 70% at the end of pregnancy (77). At the same time, HLA-G expression has been observed to be higher in term pregnancy EVT cells than in first-trimester EVTs (91). Decidual T cells have been reported to express the HLA-G receptor ILT2. In 1999, Le Gal et al. (92) found that HLA-G specifically inhibited cytolytic T cell function. M8 cells were sensitized with influenza virus peptide and the cytotoxicity of peripheral antigen-specific CD8^+^ CTL towards M8 cells was specifically decreased when M8 cells expressed HLA-G1. This inhibition could be rescued by anti-HLA-G1 mAb. In addition, Bainbridge et al. (93) reported that expression of HLA-G on C1R B-lymphocyte cells inhibited CD4^+^ T cell proliferation when peripheral blood mononuclear cells were stimulated by C1R. Furthermore, HLA-G1 was reported to induce upregulation of ILT2 and KIR2DL4 mRNA in peripheral CD4^+^ T cells when peripheral blood mononuclear cells (PBMCs) were co-cultured with HLA-G1-expressing LCL 721.221 cells (84). A proportion of peripheral CD4^+^ T cells and CD8^+^ T cells express ILT2 on their surfaces. Further, addition of anti-ILT2 mAb was observed to enhance cytotoxicity of CD8^+^ CTL towards target cells (94). Soluble ILT2 and ILT4 could competitively inhibit binding of soluble recombinant CD8αα to soluble MHC-I molecules including HLA-G1 (95). LeMaoult et al. (96) found that peripheral CD4^+^ T cells and CD8^+^ T cells were able to acquire HLA-G1 by trogocytosis from LCL 721.221 cells, which express HLA-G1. This process did not require any interaction between HLA-G1 and receptors since addition of anti-HLA-G1 mAb or anti-ILT2 mAb did not affect the trogocytosis capability. Acquired HLA-G1 inhibited proliferation of peripheral CD4^+^ T cells stimulated by IL-2 or allogeneic PBMCs. In addition, CD4^+^ T cells that acquired HLA-G1 turned into regulatory cells and inhibited activation and proliferation of autologous T cells. Furthermore, peripheral T cells could also use trogocytosis to acquire ILT2 (97). Given that EVTs express high levels of HLA-G, and that Treg cells account for a high proportion of T cells in decidua, decidual T cells may also obtain HLA-G from EVTs and transform into Treg cells. Interestingly, Tilburgs et al. (98) reported that the co-culture of T cells with EVTs could increase the percentage of CD4^+^CD25^hi^FOXP3^+^CD45RA^+^ Treg cells in a population of decidual T cells or peripheral T cells. Recently, Salvany-Celades et al. (99) identified three types of Treg cells in decidua: CD25^hi^Foxp3^+^, PD1^hi^, and TIGIT^+^ Treg cells. In their experiments, all these Treg cells suppressed proliferation of decidual CD4^+^ or CD8^+^ T cells stimulated with anti-CD3 and anti-CD28 beads. CD25^hi^Foxp3^+^, PD1^hi^ Treg cells could inhibit IFN-γ, TNF-α production of decidual CD4^+^ or CD8^+^ T cells. In addition, co-culture with EVTs was observed to increase the proportion of CD25^hi^Foxp3^+^ and PD1^hi^ Treg cells but not of TIGIT^+^ Treg cells that make up the population of CD4^+^ peripheral T cells. This process would be expected to require cell-cell contact. However, addition of blocking antibody against HLA-G had no effect on the increase of the proportion of Treg cells in the population of peripheral T cells. Whether HLA-G mediates an increase in the quantity of Treg cells in decidua remains to be investigated.

CD14^+^ macrophages constitute the second largest population of leukocytes in the decidua and express HLA-G receptors ILT2 and ILT4. Decidual macrophages in the first trimester consist of two distinct populations: CD11c^hi^ and CD11c^lo^CD209^hi^CD206^hi^. These two subsets have been shown to constitutively produce IL-6, TNF-α, and TGF-β, whereas IL-10 and MIP-1β have been shown to be mainly secreted by CD11c^hi^ decidual macrophages (100). Expression of ILT2 and ILT4 were increased in peripheral monocytes stimulated with M8 cells expressing HLA-G1 or HLA-G5 (95). In addition, HLA-G1-transfected antigen presenting cells (APC) lines could inhibit proliferation of CD4^+^ T cells, which was not mediated by the release of HLA-G1 in the medium. Further, APCs expressing HLA-G1 induced the differentiation of CD4^+^ T cells into suppressive T cells (74). The inclusion of decidual macrophages was observed to increase the percentage of Treg cells in the population of peripheral CD4^+^ T cells (99). However, it is still unclear whether HLA-G participates in immune tolerance mediated by macrophages.

DC-SIGN^+^ (CD11c^+^CD1c^+^) cells make up about 3% of decidual leukocytes (101). IL-10-producing DCs have been reported to be present in PBMCs, and are termed DC-10. DC-10 has been indicated to be able to induce a differentiation of naïve CD4^+^ T cells into IL-10-producing Tr1 cells when subjected to an ILT4/HLA-G signal. And this interaction induced the expression of HLA-G on CD4^+^ T cells (102). Guo et al. (103) found that trophoblasts express thymic stromal lymphopoietin (TSLP) and secret soluble TSLP. Decidual CD1c^+^ DCs were observed to secrete a lot of IL-10 and CCL-17 when stimulated with soluble TSLP, thereby promoting the differentiation of decidual CD4^+^ T cells into Th2 cells. In addition, decidual DCs stimulated by TSLP could induce decidual CD4^+^CD25^-^ T cells to differentiate into CD4^+^CD25^+^FOXP3^+^ Treg cells through TGF-β1 (75).

In addition, granulocytic myeloid-derived suppressor cells (GR‐MDSCs) were found to accumulate in the term placenta (104). GR-MDSCs in the peripheral blood of pregnant woman express ILT2 and ILT4. Soluble HLA-G can increase the suppressive activity of placental GR-MDSCs on T cell proliferation (105). Therefore, GR-MDSCs contribute to the formation of immune tolerance in placenta.

## HLA-G Facilitates Fetal Growth

In addition to promoting remodeling of spiral arteries and immune tolerance, HLA-G has been found to facilitate fetal growth by stimulating secretion of growth promoting factors (GPFs) in NK cells, according to recent studies (68, 106, 107).

Fu et al. (68) found that decidual NK cells in the first trimester expressed high quantities GPFs such as pleiotrophin (PTN), osteoglycin (OGN), and osteopontin (OPN) at the mRNA and protein levels. Most GPF-positive NK cells were CD49a^+^Eomes^+^ tissue resident NK (trNK) cells. However, a smaller percentage of the decidual NK cells in the first trimester from recurrent spontaneous abortion (RSA) patients were trNK cells, and these first-trimester decidual NK cells from RSA patients showed decreased secretion of GPFs, while expression of GPFs in trNK cells from patient decidua was decreased. In order to explore whether the defect of trNK cells and GPFs could affect fetal development, Fu et al. constructed a pregnancy model of NK cell knockout mice, pregnant *Nfil3^-/-^* mice. In the uteruses of pregnant *Nfil3^-/-^* mice, the number of trNK cells and the GPF levels were decreased. In addition, the average weight of fetuses from pregnant *Nfil3^-/-^* mice was decreased and the development of the embryonic skeletal system was defective. Fetal growth restriction (FGR) in pregnant *Nfil3^-/-^* mice could be rescued by transferring induced CD49a^+^ uterus-like trNK cells (108). Furthermore, pregnant GPF knockout mice showed the same fetal growth defect as did *Nfil3^-/-^* mice. CD49a^+^ uterus-like trNK cells differentiated from GPF knockout mice could reach the uterus but could not rescue the fetal development defect in pregnant *Nfil3^-/-^* mice. Moreover, injection of anti-PTN or anti-OGN antibody caused significantly reduced fetal weight. And injection of PTN could restore the fetal weight defect in pregnant *Nfil3^-/-^* mice. These findings revealed that decidual trNK cells promote fetal development by secreting growth-promoting factors.

**Figure 2:**
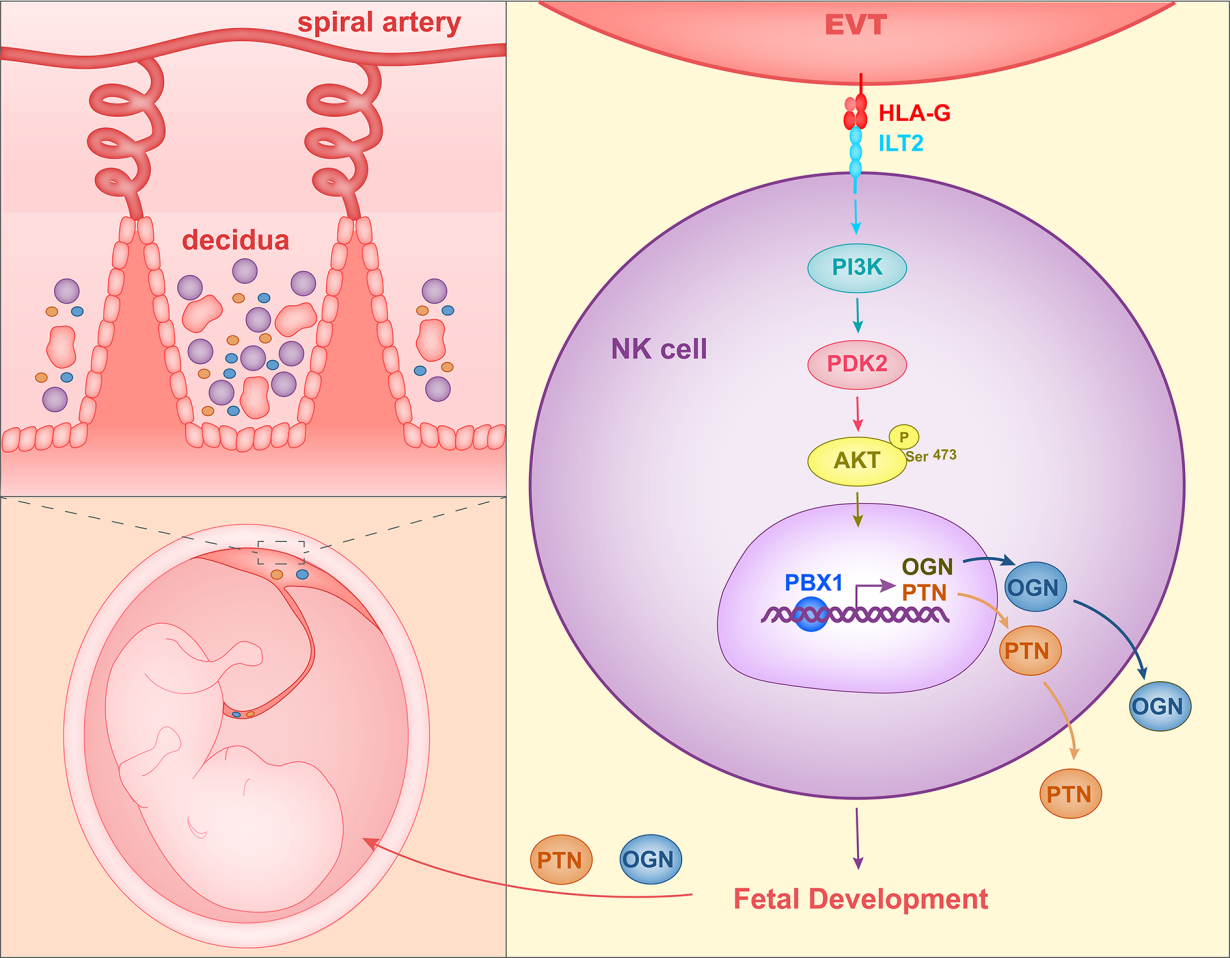
Mechanism of HLA-G interacting with decidual NK cells to promote fetal growth. HLA-G on EVTs binds to ILT2 on NK cells, activates the PI3K-AKT signal pathway and induces expression of transcription factor PBX1. PBX1 upregulates the secretion of growth-promoting factor PTN and OGN, facilitating early fetal growth.

When co-cultured with EVTs *in vitro*, decidual NK cells expressed higher levels of GPFs. Since HLA molecules on EVTs and their receptors on NK cells are important for the maternal-fetal interface, Fu et al. co-cultured decidual NK cells with LCL 721.221 cells, which expressed HLA-G or HLA-C. The co-culture assay revealed that HLA-G promote expression of PTN, OGN, and OPN in dNK cells. TrNK cells expressed HLA-G receptors ILT2 and intracellular KIR2DL4. In the co-culture assay, HLA-G antibody and ILT2 antibody reduced GPF secretion significantly in decidual NK cells stimulated by EVTs. At the same time, GPF expression levels in decidual NK cells transfected with KIR2DL4 siRNA also decreased when the NK cells were co-cultured with EVTs. Therefore, HLA-G in EVTs could promote GPF secretion in trNK cells by acting on the receptors ILT2 and KIR2DL4 (68). Zhou et al. (109) analyzed the changes of signaling pathways in decidual NK cells stimulated with EVT and found that the PI3K-Akt signaling pathway was significantly altered. Phosphorylation of AKT1 at Ser-473 and the expression of PDK2 were increased in decidual NK cells co-cultured with EVTs. In addition, transcription factor PBX1, which is already expressed in high quantities in decidual NK cells, showed even higher levels of expression in EVT-stimulated first-trimester decidual NK cells. SiRNA and phosphorylation inhibitor of AKT1 or PDK2 could reduce the levels of expression of PBX1 in decidual NK cells stimulated with EVTs. HLA-G and ILT2 blocking antibody were each shown to reduce the levels of phosphorylation of AKT1 at Ser-473 and reduce the levels of PBX1 expression in decidual NK cells co-cultured with EVTs. Therefore, HLA-G in EVTs could stimulate the PDK2-AKT1 signaling pathway and increase PBX1 expression by interacting with ILT2 in decidual NK cells. Zhou et al. also demonstrated that PBX1 could enhance the expression of growth-promoting factors PTN and OGN by directly binding to their promoters. PBX1 gene was mutant and protein level of PBX1 was reduced in decidual NK cells of RSA patients. In addition, trNK cell number and PTN and OGN levels were decreased and fetal development was impaired in mice having PBX1 knocked out specifically in NK cells. Therefore, these results indicated that HLA-G in EVTs interacts with ILT2 in decidual NK cells and activates the PDK2-AKT1 signaling pathway in NK cells, and in turn PBX1 promotes fetal growth by upregulating PTN and OGN (**Figure 2**).

Imbalance of maternal-fetal immune tolerance and fetal growth embryo development can lead to miscarriage. Plasmatic levels of soluble HLA-G, both sHLA-G1 and HLA-G5, in women with abortion was much lower than those in pregnant women. In addition, sHLA-G1 was absent in the serum of women with RSA (31). Furthermore, Nowak et al. found that women who were heterozygous in −716 HLA-G (−716 T>G SNP in the promoter region of HLA-G) had a lower possibility of spontaneous miscarriage (110). In the promoter region of HLA-G, -1573 T>C SNP and -1746 C>A SNP were also reported to be associated with RSA (33). Therefore, studying the role of HLA-G during pregnancy could be beneficial to the understanding of RSA.

## Conclusions

**Figure 3:**
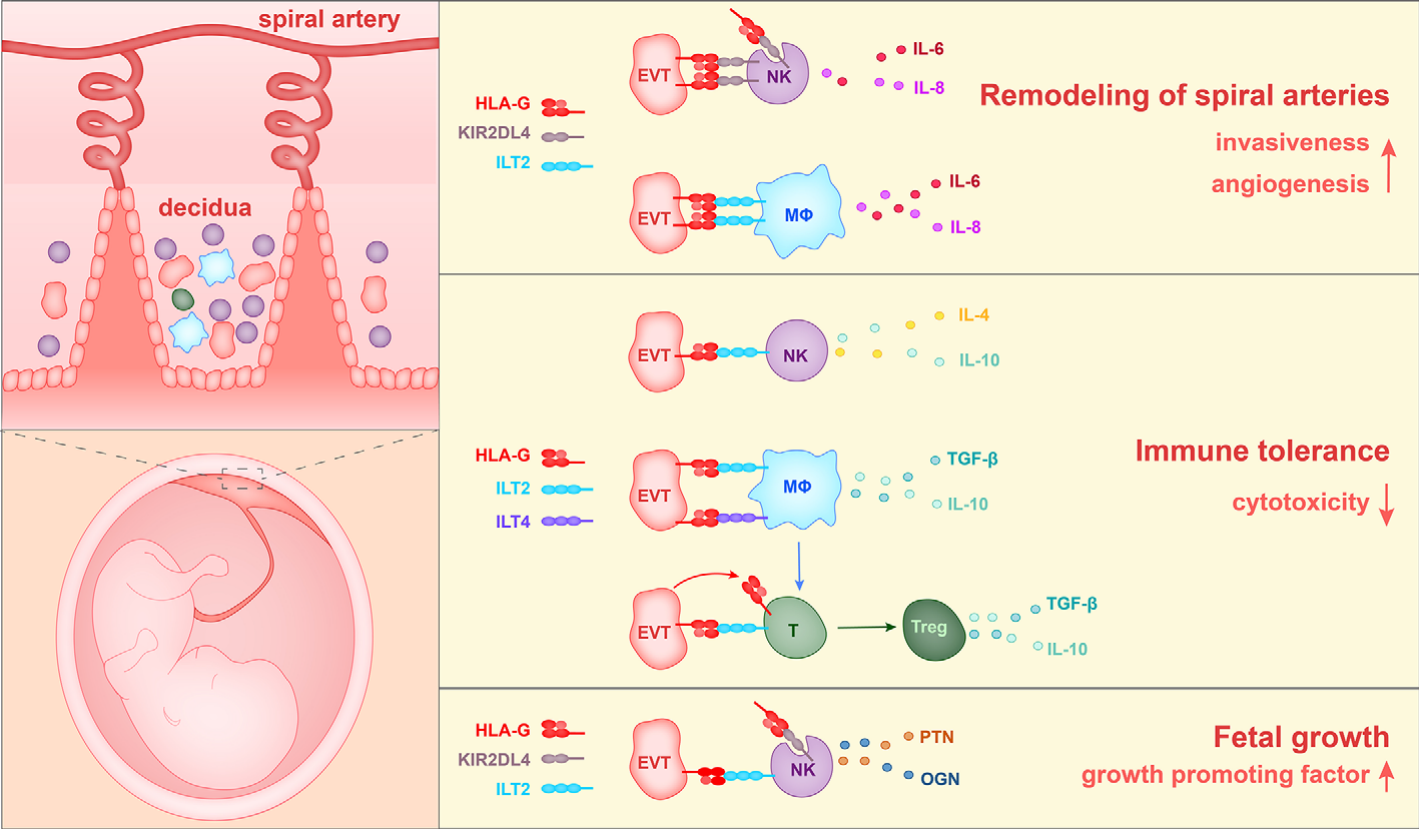
The currently identified roles of HLA-G in the pregnancy microenvironment. 1) HLA-G on extravillous trophoblasts (EVTs) binds to KIR2DL4 on NK cells or ILT2 on macrophages, and in this way stimulates the production of IL-6, IL-8, and VEGFα. Soluble HLA-G binds to KIR2DL4 on NK cells and induces the production of IL-6 and IL-8. Thus HLA-G promotes vascular permeability, angiogenesis and EVT invasiveness, and thereby participates in the remodeling of spiral arteries. 2) HLA-G on EVTs binds to ILT2 on NK cells, ILT2 and ILT4 on macrophages, and ILT2 on Treg cells, and reduces levels of cytotoxicity towards fetal tissues. 3) HLA-G on EVTs binds to ILT2 and KIR2DL4 on NK cells and promotes the secretion of growth-promoting factors PTN and OGN, thereby facilitating fetal growth.

To summarize the above reports, three roles of HLA-G have been found during pregnancy. HLA-G interacts with ILT2 and KIR2DL4 on macrophages and NK cells to enhance the production of proangiogenic cytokines and to enhance the EVT invasion of decidua, thereby promoting spiral artery remodeling. In addition, HLA-G binds to ILT2, ILT4, and KIR2DL4 on NK cells, T cells and macrophages, inhibits the cytotoxicity of NK cells and CD8^+^ T cells, and causes an increase in the percentage of Treg cells in the population, and thereby contributes to immune tolerance. Furthermore, HLA-G on EVTs could induce the production of growth-promoting factors by decidual NK cells, thereby regulating fetal growth (**Figure 3**).

Since mice do not express HLA-G, samples are difficult to obtain and *in vitro* culture cannot be maintained for a long time, thus, it is difficult to study the function and mechanism of HLA-G without functional experimental models. Interestingly, Turco et al. (111) constructed, from first-trimester villi, long-lasting genetically stable trophoblast organoids that could differentiate into EVTs. Experiments deploying this system are expected to be used to further investigate the role and mechanism of HLA-G in pregnancy and to test the current models.

HLA-G has been reported to be abnormally expressed in many kinds of tumor tissues and has been detected in the plasma of cancer patients. In addition, the expression of HLA-G was found related to the outcome in various tumors (112). Since HLA-G is abnormally and specifically expressed in tumor tissues, it may represent a checkpoint in tumor immunology (113). At the maternal-fetal interface, HLA-G has been found to inhibit the cytotoxicity of T cells and NK cells and increase the proportion of Treg cells. It may also perform similar functions in tumor microenvironment. Therefore, the roles of HLA-G in pregnancy may provide clues for further understanding of tumor immunology.

## Author Contributions

All authors contributed to the article and approved the submitted version. XX drafted the manuscript and figures. HW and YZ edited/reviewed the article.

## Funding

This work was supported by the key project of the National Key Research and Development Program of China (#2018YFC1003900) and the National Natural Science Foundation of China (#31900662).

## Conflict of Interest

The authors declare that the research was conducted in the absence of any commercial or financial relationships that could be construed as a potential conflict of interest.
